# The Clinical Impact of the C_0_/D Ratio and the CYP3A5 Genotype on Outcome in Tacrolimus Treated Kidney Transplant Recipients

**DOI:** 10.3389/fphar.2020.01142

**Published:** 2020-07-31

**Authors:** Teun van Gelder, Soufian Meziyerh, Jesse J. Swen, Aiko P. J. de Vries, Dirk Jan A. R. Moes

**Affiliations:** ^1^ Department of Clinical Pharmacy & Toxicology, Leiden University Medical Center, Leiden, Netherlands; ^2^ Department of Internal Medicine, Division of Nephrology, Leiden University Medical Center, Leiden, Netherlands; ^3^ Leiden Transplant Center, Leiden University Medical Center, Leiden, Netherlands

**Keywords:** tacrolimus, transplantation, kidney, CYP3A5, pharmacogenetics

## Abstract

Tacrolimus is metabolized by CYP3A4 and CYP3A5 enzymes. Patients expressing CYP3A5 (in Caucasian patients about 15% of the population but more frequent in African Americans and Asians) have a dose requirement that is around 50% higher than non-expressers to reach the target concentration. CYP3A5 expressers can be considered fast metabolizers. The trough concentration/dose (C_0_/D) ratio of tacrolimus has recently been proposed as a prognostic marker for poor outcome after kidney transplantation. Patients with a low C_0_/D ratio (also referred to as fast metabolizers) seem to have more tacrolimus-related nephrotoxicity, more BK-viremia, and a lower graft survival. At first sight, the expression of CYP3A5 and a low C_0_/D ratio seem to be overlapping factors, both pointing towards patients in whom a higher tacrolimus dose is needed to reach the tacrolimus target concentration. However, there are important differences, and these differences may explain why the impact of the C_0_/D ratio on long term outcome is stronger than for CYP3A5 genotype status. Patients with a low C_0_/D ratio require a high tacrolimus dose and are exposed to high tacrolimus peak concentrations. The higher peak exposure to tacrolimus (and/or its metabolites) may explain the higher incidence of nephrotoxicity, BK-viremia and graft loss. A potential confounder is the concurrent maintenance treatment of corticosteroids, as steroids are sometimes continued in patients at high immunological risk. Steroids induce the metabolism of tacrolimus *via* pregnane X receptor mediated increased CYP3A4 expression, resulting in lower tacrolimus C_0_/D ratio in high risk patients. Also non-adherence may result in lower C_0_/D ratio which is also associated with poor outcome. The C_0_/D ratio of tacrolimus does seem to identify a group of patients with increased risk of poor outcome after kidney transplantation. Our recommendation is to monitor tacrolimus peak concentrations in these patients, and if these are high then target slightly lower pre-dose concentrations. Another possibility would be to switch to a prolonged release formulation or to dose the drug more frequently, in smaller doses, to avoid high peak concentrations.

## Introduction

Tacrolimus is the first choice calcineurin inhibitor (CNI) in kidney transplant patients. Maintaining the tacrolimus exposure within the therapeutic window is considered to be essential to prevent the development of cellular and antibody-mediated rejections and to minimize drug-related toxicity.

The pharmacokinetics (PK) of tacrolimus is characterized by poor and highly variable oral bioavailability (mean 25%, range 5–90%) (Shuker et al., 2015). Also drug–drug interactions, epigenetic changes in the expression of metabolizing enzymes and patient adherence contribute to a large inter-patient and intra-patient variability in tacrolimus exposure (Vanhove et al., 2016). An important part of the inter-patient variability is explained by the presence of a single nucleotide polymorphism (SNP) in the gene encoding for the cytochrome P450 (CYP) 3A5 enzyme (6986A>G). Patients expressing CYP3A5 (those carrying the A nucleotide, defined as the *1 allele) have a dose requirement that is around 50% higher than non-expressers (those homozygous for the G nucleotide, defined as the *3 allele) (Hesselink et al., 2014). Also the CYP3A4 gene carries a SNP (SNP in intron 6 (rs35599367C>T)) that is significantly associated with the tacrolimus dose requirement, but to a lesser degree than the CYP3A5 gene polymorphism (Elens et al., 2011). Genome wide association studies have shown that there are no other common single genetic variants aside from the CYP3A gene that significantly influences the tacrolimus PK (Oetting et al., 2018; Oetting et al., 2019).

Intra-patient variability in tacrolimus exposure is easily identified by repetitive measurement of drug concentrations in patients on maintenance treatment. Ten years ago Borra et al. demonstrated that patients with high intra-patient variability more often reached a composite endpoint consisting of graft loss, biopsy-proven chronic allograft nephropathy, and ‘doubling in plasma creatinine concentration in the period between t = 12 months post-transplantation and last follow-up’ (Borra et al., 2010). A high intra-patient variability in tacrolimus exposure is now recognized as a predictor of poor clinical outcome (van Gelder, 2014; Mendoza Rojas et al., 2019). Rodrigo et al. found that a higher intra-patient variability was independently related to development of donor-specific antibodies and graft-loss (Rodrigo et al., 2016). Identification of patients with a high intra-patient variability is therefore important as it is a modifiable risk factor, and interventions may improve long-term outcomes (Neuberger et al., 2017).

## Concentration/Dose (C_0_/D) Ratio and Outcome

More recently the concentration/dose (C_0_/D) ratio of tacrolimus has also been proposed as a prognostic marker for poor outcome. The C_0_/D ratio can be calculated by dividing the tacrolimus pre-dose concentration (C_0_) by the corresponding daily tacrolimus dose (D).

$$\mathrm{Formula}:\;C_{0}/D\;\mathrm{ratio}=\mathrm{tacrolimus}\;\mathrm{trough}\;\mathrm{concentration}\;(\mathrm{ng}/\mathrm{mL})/\mathrm{daily}\;\mathrm{dose}\;(\mathrm{mg})$$

Thölking et al. were the first to hypothesize that the metabolization rate of tacrolimus, expressed as the C_0_/D ratio, would be a prognostic factor of clinical outcome (Thölking et al., 2014). The mean of the C_0_/D ratios calculated at months 1, 3, and 6 after renal transplantation was used to categorize patients as fast, intermediate, and slow metabolizers. The incidence of T cell-mediated rejection or antibody-mediated rejection was not related to the tacrolimus metabolizer status, but in the group of fast metabolizers more often CNI nephrotoxicity (p = 0.015) and BK-virus associated nephropathy (p = 0.024) were observed. They concluded that the tacrolimus C_0_/D ratio is a simple and inexpensive tool to identify patients at risk for the development of CNI nephrotoxicity or BK nephropathy. Moreover, a C_0_/D ratio < 1.05 was also associated with a higher mortality in a 24 months follow-up. In a five-year follow-up study from the same group the patient survival was noticeably reduced in fast metabolizers as compared to intermediate/slow metabolizers (89.9 *vs.* 95.3%, log-rank p = 0.036), and in a Cox regression analysis fast metabolizer status was an independent predictor of both graft and patient survival (Schütte-Nütgen et al., 2019). The suggested intervention could be to switch fast Tac metabolizers from tacrolimus based therapy to treatment with a mammalian target of rapamycin inhibitor (mTORi) or cyclosporine, but there is no evidence that this intervention does improve long term outcome. Although the authors do acknowledge that tacrolimus metabolism is also related to the CYP3A5 genotype, they claim that the prognostic value of the C_0_/D ratio is stronger than that of the genotype.

In a subsequent study, patients were categorized into three metabolizer groups based on the same cut-off values. Patients with a tacrolimus C_0_/D ratio < 1.05 ng/ml/mg were characterized as fast metabolizers, patients with a C_0_/D ratio of 1.05–1.54 ng/ml/mg as intermediate metabolizers, and those with a C_0_/D ratio ≥ 1.55 ng/ml/mg were defined as slow metabolizers (Thölking et al., 2016). Also in this study a fast tacrolimus metabolism was associated with increased risk of BK viremia. The potential explanation for the effect of the metabolizer status on outcome is that in patients with a faster metabolism the drug dose required to reach the target tacrolimus trough concentration is higher. As a result, the tacrolimus peak levels in the first hours after oral administration are higher. Evidence for this hypothesis was obtained in an additional study in 56 renal transplant recipients, in whom the tacrolimus concentrations 2 h after drug intake (C_2_) in patients with a low C_0_/D ratio (high metabolizers) were increased compared to the other patients (20.2 ± 10.3 ng/ml *vs.* 9.8 ± 4.2 ng/ml, respectively; p = 0.004) (Thölking et al., 2019). In daily practice most centers only monitor pre-dose tacrolimus concentrations, and the higher peak levels often go unnoticed.

A Polish group recently also reported impaired outcome data in patients with a low C_0_/D ratio from a large group of 571 renal transplant patients (Kwiatkowska et al., 2019). In these patients the C_0_/D ratio was calculated at their most recent out-patient appointment (mean time after transplantation = 84 months), and this ratio was then correlated with the change in renal function from transplantation to last follow-up. Also in this study a higher metabolization status was associated with a significantly greater drop in the eGFR. In a smaller study, also from Poland, the highly significant relationship between C_0_/D ratio at 6 months and kidney function 2 years after transplantation was confirmed in a linear regression model (p = 0.007) (Nowicka et al., 2019).

In 2020 Jouve et al. published a retrospective study on more than 1,000 kidney transplant patients treated with tacrolimus and with more than 1 year follow-up (Jouve et al., 2020). This study was called the TOMATO study, which stands for TacrOlimus MetAbolization in kidney TransplantatiOn. In a multivariate analysis the C_0_/D ratio at month 3 and month 6 proved to be independent of early predictors of death-censored kidney graft survival. The authors stressed the importance of mechanistic studies to understand how the C_0_/D ratio causes its effect.

Taber et al. studied the impact of tacrolimus pharmacokinetics in African Americans (AAs) (Taber et al., 2015). In AAs with sub-therapeutic tacrolimus concentrations, the incidence of acute cellular rejection and of antibody-mediated rejection was increased. But also patients who did achieve therapeutic tacrolimus concentrations were at an increased risk of developing interstitial fibrosis and tubular atrophy (IF/TA), reflecting nephrotoxicity. Most likely, in these AA patients therapeutic tacrolimus concentrations are reached at the cost of high drug doses, and the high peak concentrations inherent to these dosages result in more nephrotoxicity.

A faster metabolization of tacrolimus will result in higher concentrations of tacrolimus metabolites. These metabolites may accumulate in the blood and or renal tubular cells and cause nephrotoxicity. Compared to nonexpressers, the CYP3A5 expressers have a 2.0- to 2.7-fold higher metabolite/parent AUC ratio for these metabolites (Zheng et al., 2012). As several immunoassays suffer from cross-reactivity of these metabolites (without reporting a concentration of the individual metabolites), the mean of the tacrolimus concentrations measured with these immunoassays is higher than if measured with mass spectrometry based assays (Akamine et al., 2018). A survey in 2015 showed that for TDM of tacrolimus 53% of the laboratories used LC–MS/MS and 47% immunoassays (n = 72) (Christians et al., 2015). Accumulation of tacrolimus metabolites in the blood may go unnoticed if TDM is done exclusively on the basis of LC–MS/MS.

## CYP3A5 Genotype and C_0_/D Ratio

As already mentioned, there are multiple studies that show that renal transplant patients who express the CYP3A5 enzyme need a higher dose to reach the target tacrolimus concentration compared to non-expressers (Hesselink et al., 2003; Thervet et al., 2003; Haufroid et al., 2004). Thus, CYP3A5 expressers in general have a low C_0_/D ratio. In a Caucasian population the prevalence of patients expressing CYP3A5 is low (10%) (van Schaik et al., 2002). In contrast, in patients from African descent about 40–50% express CYP3A5, and in Asian patients the prevalence of CYP3A5 expressers is even higher (50–70%) (Andrews et al., 2016).

In most centers the starting dose of tacrolimus is based on body weight. Standard tacrolimus dosing is 0.2 mg/kg bodyweight, divided in two doses. The guideline of the Clinical Pharmacogenetics Implementation Consortium (CPIC) recommends to increase the starting dose in CYP3A5 expressers 1.5–2 fold, but not to exceed 0.3 mg/kg/day (Birdwell et al., 2015). The higher starting dose is meant to avoid early underexposure to tacrolimus in the first days after transplantation.

## CYP3A5 and Outcome

In 1994 an analysis was published on differences in outcome of black and white patients after kidney transplantation based on a large dataset from the United Network for Organ Sharing (UNOS). An impaired graft survival in black patients was found. The difference in outcome was attributed to poor socioeconomic factors and reduced access to health care in African Americans and to poor HLA-matching (Koyama et al., 1994). In this study CYP3A5 genotype data were not available. A French study demonstrated that ethnic origin did not affect outcome after renal transplantation in France, and it was suggested that the poor results of renal transplantation in patients of African origin in the US could be improved with universal immunosuppressive drug coverage (Pallet et al., 2005). Also in this French study CYP3A5 genotype data were not available.

In a meta-analysis Rojas found that the CYP3A5 expresser genotype might be associated with a higher risk of acute rejection (OR 1.32, 95% CI 1.02–1.71) and a trend towards more chronic nephrotoxicity (OR 1.81, 95% CI 0.89–3.68) (Rojas et al., 2015). However, a large-scale genome-wide association study did not identify strong donor or recipient genetic predictors of allograft survival or renal function outside the HLA region (Hernandez-Fuentes et al., 2018; Stapleton et al., 2019). Also a candidate gene association study of allograft loss in renal transplant recipients CYP3A5 was not related to outcome (Woillard et al., 2018).

In a recently published meta-analysis, the effect-size of CYP3A5 polymorphism on the risk of rejection was estimated in Asian and European kidney transplant populations (Khan et al., 2020). In European populations no significant association was found with rejection episodes between expressers and nonexpressers (OR: 1.12; p = 0.47). However, in Asian patients a higher risk of rejection was found after follow-up of 3 years post-transplantation (OR: 1.68; p < 0.05), respectively. An explanation for this difference, other than a higher prevalence of CYP3A5 expressers in Asian populations could not be given. Data on longer term follow-up are scarce, and this higher risk estimate would need confirmation in larger datasets.

## C_0_/D Ratio as Prognostic Factor for Outcome

In daily practice tacrolimus pre-dose concentrations are being monitored, and routinely monitoring tacrolimus peak concentrations or AUC is unusual since it is more cumbersome both for patients and clinicians. Whether or not the patient needs a high tacrolimus dose to reach the target concentration is not taken into account. However, patients who need a high tacrolimus dose may be exposed to substantially higher tacrolimus peak concentrations and tacrolimus-AUC compared to patients who reach the same pre-dose concentrations with lower dosages. A study of 46 African Americans, of whom 35 were CYP3A5-expresser, showed that the tacrolimus pre-dose concentrations were similar in CYP3A5-expressers and non-expressers (6.26 and 6.24 ng/ml respectively) (Trofe-Clark et al., 2018). In the expressers group a much higher tacrolimus dose was required to reach these pre-dose concentrations (10.1 *vs* 6.3 mg/day), and as a result the C_max_ was also higher in the expressers (25.5 ng/ml and 19.5 ng/ml respectively; p = 0.04). The higher peaks and higher AUC may lead to chronic nephrotoxicity and ultimately contribute to graft loss. In a study on chronic irreversible drug-induced nephrotoxicity Kuypers et al. also found that especially patients with a high early tacrolimus dose requirement developed this form of toxicity (Kuypers et al., 2010). In their study this was predominantly but not exclusively encountered in CYP3A5 expressers.

At present tacrolimus is being dosed based on trough concentrations in the vast majority of centers. There is limited experience with dosing tacrolimus based on AUC. In Leiden the tacrolimus target AUC_0–12h_ for maintenance treatment (>6 months post-transplantation) is 80 ug*h/L. Based on a pharmacokinetic model the upper threshold of this target relates to a peak concentration of 22 ng/ml (Scholten et al., 2005). In case of high peak concentrations and high AUC the tacrolimus dose can be reduced. If peaks are high but AUC is within the target, then dose could be divided into smaller portions (from twice daily to three times daily), but this will negatively affect adherence. An alternative option would be to change from a twice daily immediate release formulation to a once daily prolonged release formulation. Besides avoiding high peaks this will also improve adherence. Another option would be to switch to mTOR-inhibitors as suggested by Thölking et al. (Schütte-Nütgen et al., 2019). There are no studies available that show that either of these options is beneficial.

At first sight the expression of CYP3A5 and a low C_0_/D ratio seem to be overlapping factors, both pointing towards patients in whom a higher tacrolimus dose is needed to reach the tacrolimus target concentration. However, there are important differences, and these differences may explain why the impact of the C_0_/D ration on long term outcome is stronger than for CYP3A5 genotype status.

First of all, on average the CYP3A5 genotype does lead to a higher dose requirement, but there is considerable overlap between expressers and non-expressers (Thervet et al., 2003). Some of the CYP3A5 expressers do not have a fast metabolizer phenotype, and they reach target concentrations with conventional tacrolimus doses. In these patients there is not a high peak concentration after drug intake. In contrast, patients with a low C_0_/D ratio all have a fast metabolizer phenotype and invariably need high doses of tacrolimus. As a result, the impact of the C_0_/D ratio may be stronger than the CYP3A5 genotype.

Another factor that may impact on the prognostic significance of the C_0_/D ratio is the use of corticosteroids in patients at increased risk of rejection. For patients at low-immunological risk the avoidance, or early withdrawal of steroids, is well tolerated, but in patients with higher risk this may lead to an increased incidence of acute cellular rejections or late antibody medicated rejections (Pascual, 2011). Therefore, continued exposure to steroids may be linked to a higher immunological risk. As steroids are known to induce CYP3A enzymes the tacrolimus metabolism is faster in patients treated with steroids, and C_0_/D ratio is lower (Hesselink et al., 2003; van Duijnhoven et al., 2003). Steroid use may thus be a confounder by indication, affecting the C_0_/D ratio. However, in the recently published TOMATO-study the C_0_/D ratio was independently associated with death-censored kidney-graft survival, even when corrected for continued corticosteroid use (Jouve et al., 2020).

An important limitation is that the number of studies on this topic is still limited and that several of these studies have been published by one center (Thölking et al.). In order to be more certain on the clinical relevance of the C_0_/D ratio it would be good if more well documented analyses in larger data sets, including correction for potential confounding factors such as ethnicity, steroid use, and intra-patient variability, would be performed. Furthermore, interventions such as dose reductions and switching to prolonged release formulations should ideally be tested in prospective controlled studies. Novel developments in therapeutic drug monitoring, including home-based dried blood spot (DBS) sampling, offer the potential to facilitate repetitive large scale AUC assessment as an alternative to the conventional TDM sampling at the (out-patient) clinic. Liquid chromatography-tandem mass spectrometry (LC-MS/MS) assays have been developed to quantify tacrolimus in DBS samples (Veenhof et al., 2017; Zwart et al., 2018), and at home sampling has been shown to be feasible in the kidney transplant population. Adherence to immunosuppressive therapy has been shown to decrease over time (Massey et al., 2015). Furthermore, non-adherence is a strong predictor for poor outcome and is related to development of donor specific antibodies and a higher risk of rejection (Takemoto et al., 2007). Patients who do not take (part of) their daily drug dose seemingly have a fast metabolism and a low C_0_/D ratio. In these patients the poor outcome is not due to the faster metabolism, or due to high peak concentrations but to non-adherence.

## Conclusion

The C_0_/D ratio of tacrolimus does seem to identify a group of patients with increased risk of poor outcome after kidney transplantation. Our recommendation is to check tacrolimus peak concentrations in these patients, and if these are high then target for slightly lower pre-dose concentrations. Assessment of limited sampling AUC can assist in finetuning the tacrolimus dose.

## Author Contributions

TG and DM developed the idea for this manuscript. The first draft version was written by TG. All authors contributed to the article and approved the submitted version.

## Conflict of Interest****

In the last 3 years TG has received lecture fees and study grants from Chiesi and Astellas, in addition to consulting fees from Roche Diagnostics, Vitaeris, Astellas, Aurinia Pharma and Novartis. In all cases money has been transferred to hospital accounts, and none has been paid to his personal bank accounts. TG does not have employment or stock ownership at any of these companies, and neither does he have patents or patent applications.

The remaining authors declare that the research was conducted in the absence of any commercial or financial relationships that could be construed as a potential conflict of interest. 
